# Real-time on-machine observations close to interelectrode gap in a tool-based hybrid laser-electrochemical micromachining process

**DOI:** 10.1038/s41598-020-73821-7

**Published:** 2020-10-08

**Authors:** Krishna Kumar Saxena, Xiaolei Chen, Maria Rosaria Vetrano, Jun Qian, Dominiek Reynaerts

**Affiliations:** 1 Micro- & Precision Engineering Group, Manufacturing Processes and Systems, Department of Mechanical Engineering, KU Leuven, Leuven, Belgium; 2 Division of Applied Mechanics and Energy Conversion, Heat and Mass Transfer Group, Department of Mechanical Engineering, KU Leuven, Leuven, Belgium; 3 Member Flanders Make, Leuven, Belgium; 4 School of Electromechanical Engineering, Guangdong University of Technology, Guangzhou, China; 5 Guangzhou Key Laboratory of Nontraditional Machining and Equipment, Guangzhou, China

**Keywords:** Nanoscience and technology, Mechanical engineering

## Abstract

A tool-based hybrid laser-electrochemical micromachining process involves concurrent application of two process energies i.e. electrochemical and laser in the same machining zone by means of a hybrid tool which serves as an ECM tool as well as a multimode waveguide. It is a relatively novel process finding applications in defect-free machining of difficult-to-cut materials without affecting their microstructure. In order to understand the physical phenomena occurring during this process, in-situ observations are required. Therefore, in this work, a real time observation was carried out of a novel tool-based hybrid laser electrochemical micromachining process. A combination of high-speed imaging and Large Scale Particle Image Velocimetry (LSPIV) was used to visualize the tool-based hybrid laser-ECM process in real time. It also allowed to carry out experimental investigations on the by-products and bubble generation which have a direct effect on process performance in terms of accuracy and efficiency. The real-time on-machine observations are unique of its kind and they will facilitate the understanding of underlying mechanisms governing this hybrid laser-electrochemical micromachining process. This will ultimately help in improving the quality of parts manufactured. This research is also a step forward towards making these physics-based hybrid processes deterministic by employing high-speed imaging in a closed loop control.

## Introduction

Electrochemical micromachining^1^ (ECM) is a non-traditional and non-contact machining process which has capability to machine difficult-to-cut materials such as titanium^2^, bulk metallic glasses^3^, superalloys^4,5^, etc. without compromising on material properties and with the added advantage of no process related tool-wear. As the process involves material removal by anodic dissolution^6^, it generates high quality and defect free surfaces as compared to laser micromachining and electro-discharge micromachining. To further advance the process capabilities of electrochemical micromachining, hybridization with other processes is being researched^6^. Hybrid micromachining^7^ involves simultaneous or sequential application of two or more time-dependent^8^ processes in the same machining zone. Various hybrid micromachining processes involving electrochemical micromachining as a primary energy source; are under development including electrochemical grinding^4^, electrochemical-discharge machining, laser-electrochemical machining^9–11^ and ultrasonic assisted electrochemical machining^12^. The hybrid laser-electrochemical micromachining process is based on simultaneous application of laser and electrochemical processes in the same machining zone. The material removal mechanisms can be laser assisted electrochemical removal or combined laser-electrochemical removal depending on the laser fluence and process-timing available on the workpiece side. This hybridization of laser with ECM^13^ offers several advantages such as: (1) a laser induced temperature rise increasing the kinetics of electrochemical reactions and leading to increased current densities^9,14^, (2) a laser induced structural weakening of passivating layer (e.g. in titanium machining^2^) in case of electrochemical machining with passivating electrolytes; and (3), gentle material processing^15^ and machining of advanced materials with conductivity variations. It has been reported that the laser induced thermal field results in a reduction of passivation layer^9,13^ (O_2_ content) on STAVAX mould steel surface by nearly 50% as compared to the ECM process. Nearly 25% reduction in passivation layer was observed for Ti6Al4V with laser-ECM as compared to the ECM process However, this was not the case with WC and NbC where there was no difference in the oxygen content of the surface with ECM and laser-ECM as observed from EDX analysis. The weakening of oxide layer is also seen up to a specific laser pulse energy (45 µJ, in the reported experimental configuration^13^). When comparing laser-ECM to ECM for the given experimental parameters, a 6.03%, 7% and 9.5% rise in average volumetric MRR (material removal rate) has been observed for effective laser pulse energies of 10, 36 and 60 μJ, respectively. The MRR starts to drop with further increase in laser pulse energy beyond 60 μJ^13^.

Several physical phenomena govern the quality of electrochemically machined workpieces, including hydrogen bubble generation, oxygen gas generation, workpiece passivation, heat generation, generation of reaction-byproducts. Since, these phenomena occur in and around a machining gap of less than 100 µm, it is difficult to study the mechanism of the process experimentally owing to technological limitations. For the hybrid laser-electrochemical micromachining process, the conditions in the inter-electrode gap are even more complicated and need further investigation. Evaluation of physical phenomena close to the machining gap is critical for understanding the underlying process mechanisms especially in hybrid processes. A better visualization of process in the machining zone^16^ helps in achieving deterministic material removal leading to high quality products. Some attempts have been made by Kunieda et al.^17^ to visualize inter-electrode gap phenomena using a transparent electrode. Klocke et al.^18^ visualized gas evolution and temperature in the ECM process using a dedicated setup. In the work of Natsu et al.^19^, visualization and analysis of bubbles in the electro-discharge machining process were carried out. Julfekar et al.^20^ investigated the effect of tool-electrode surface roughness on the gas film thickness and dimensional overcut during the electrochemical discharge machining (ECDM) process. It was observed that a higher roughness of the tool-electrode resulted in a thicker gas film and hence higher overcut and vice-versa. The studies mentioned above have achieved significant understanding of electro-discharge as well as electrochemical machining. However, there are few studies available for hybrid micromachining processes. The existing studies are conducted on proof-of-concept test stands where the conditions are totally different than those on the actual machine. In light of the above, this work presents real-time on-machine observations during machining with a tool-based hybrid laser-electrochemical micromachining process^13^. A combination of high speed imaging and particle image velocimetry was used to understand by-product generation, bubble generation, and electrolyte flow behavior close to the machining zone. By further advancements in tool-based hybrid laser-ECM process in combination with the fundamental knowledge generated from real-time observations, this technology can create a technological breakthrough in scalable micromachining of advanced functional materials. For manufacturing industries, this research is a first step towards making these hybrid processes deterministic by employing high speed imaging in closed loop control.

## Results and discussions

### Principle of tool-based hybrid laser-electrochemical micromachining

**Figure 1:**
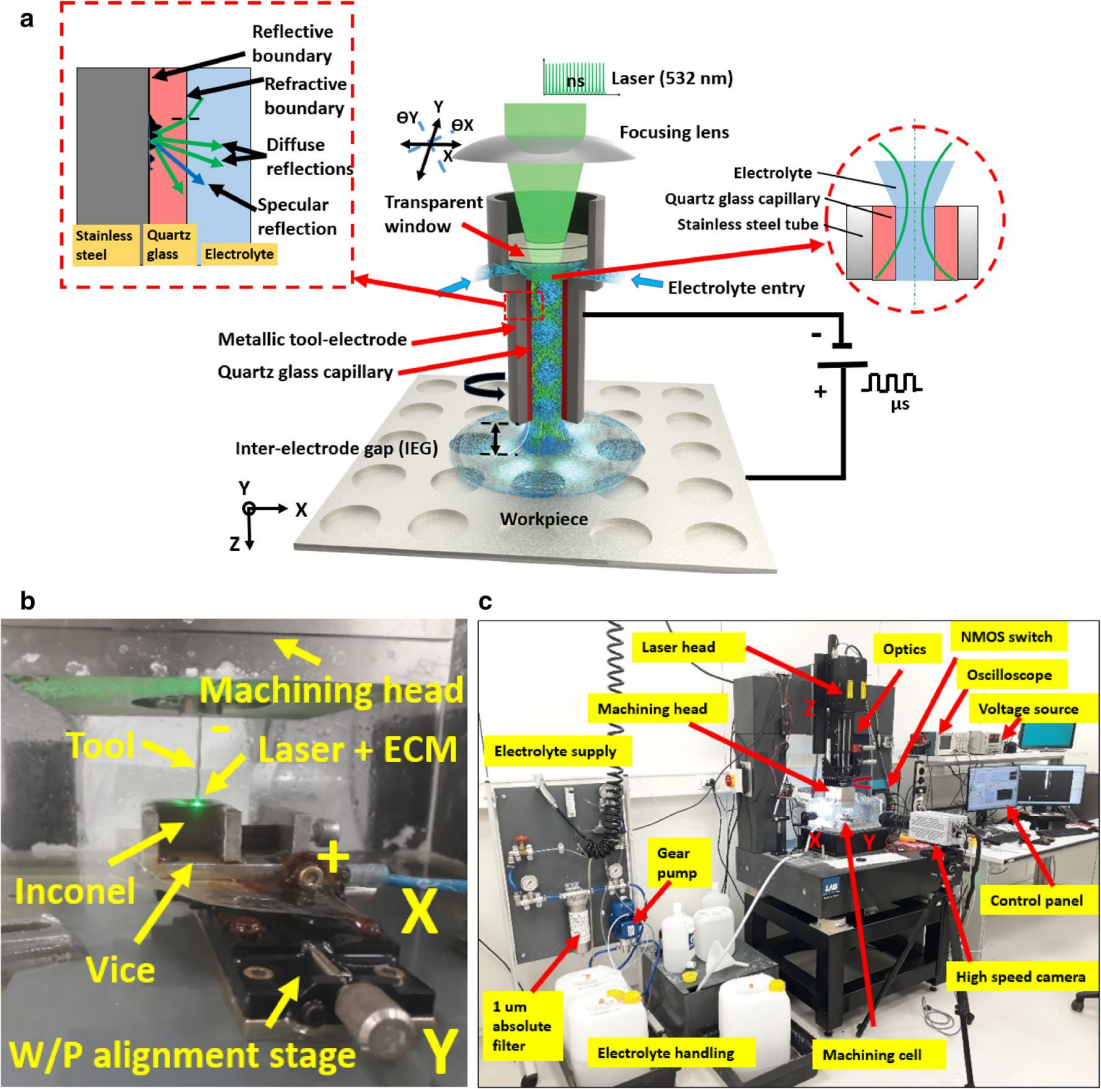
Process schematic of a tool-based hybrid laser-electrochemical micromachining process (top). A snapshot of the actual setup during machining (bottom). The process comprises of two process energies along same machining axis i.e. electrochemical machining process and a laser source^13^. (Reproduced from—Krishna Kumar Saxena, Jun Qian, Dominiek Reynaerts, “A tool-based hybrid laser-electrochemical micromachining technology: experimental investigations and synergistic effects”, International Journal of Machine Tools and Manufacture, vol. 155, 2020.)

Figure 1a,b shows a schematic illustrating the principle of a tool-based hybrid laser-electrochemical micromachining process^13^. Figure 1c depicts the hybrid machine tool prototype with major peripherals developed at KU Leuven. The system comprises a ns pulsed laser source (532 nm) and a µs pulsed voltage source for the ECM process. Both laser and ECM are applied concurrently and on the same machining axis by means of a hybrid tool^21^ which acts as both an ECM tool and a multimode waveguide for the laser. In the tool-based laser-ECM process described in this work, the laser is focused close to the inlet of tubular electrode after which it propagates further by means of multiple reflections inside the tool-electrode. The green laser is implemented as it has minimal absorption in pure water as compared to IR and UV lasers. The absorption coefficient reported for green laser (532 nm) in pure water is 0.045/m^22^ which is much less than what has been reported for IR and UV lasers. In our experimental results, it was observed that with a green laser and with 100 g*/*l* aq.* NaNO_3_, nearly 96% of the applied power is available after traversing through a 70 mm high electrolyte column^13,21^. In case of the electrolyte used here, power loss may also arise from scattering of light interacting with suspended salt particles. To overcome this issue, a 1 µm absolute filter was used in the electrolyte circuit^21^.

**Figure 2:**
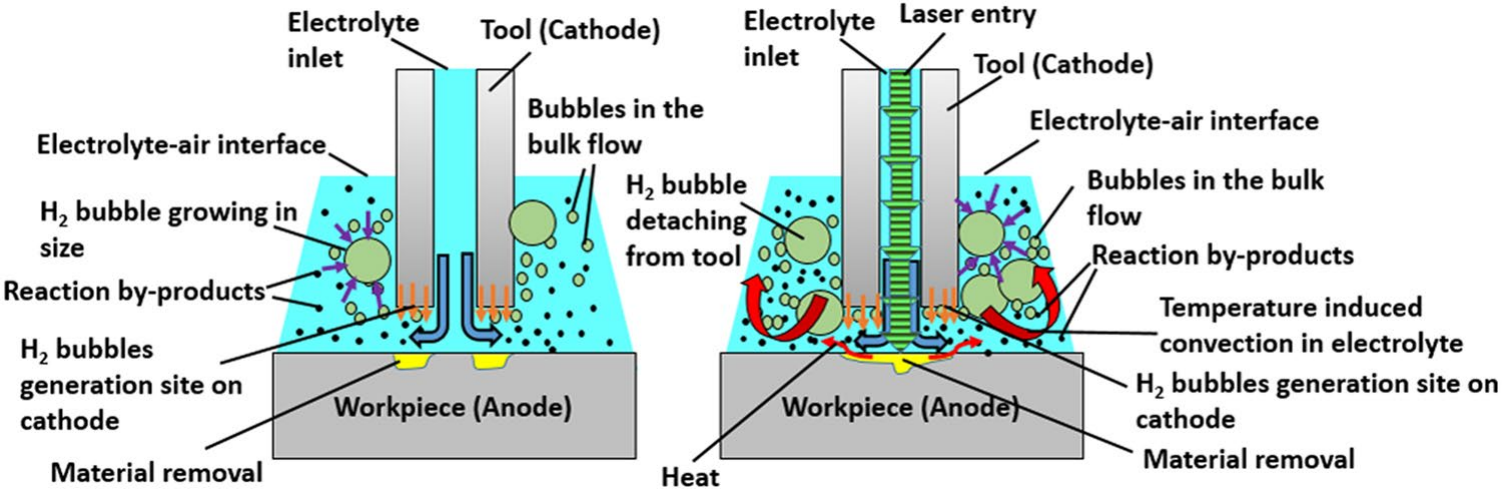
Schematic representation of the microscopic phenomenon in and around interelectrode gap in ECM process (left) and hybrid laser-ECM process (right). It is clear that in hybrid laser-ECM, the temperature induced rise in kinetics gives rise to accelerated generation of by-products and hydrogen gas bubbles.

Details on the machining procedure are mentioned in the methods section. Figure 2 illustrates the microscopic phenomena occurring during electrochemical machining as well as laser-electrochemical machining. During the electrochemical machining process, the tool is assigned negative polarity and the workpiece is connected to the positive terminal of the pulsed power supply. Electrolyte is supplied under pressure (~ 3 bar) and is delivered into the interelectrode gap (~ 20 µm) through the tubular hybrid tool. Due to the applied external voltage, material dissolution takes place according to Faraday’s law. As a side product of electrochemical reactions, hydrogen gas is produced at the cathode (tool) and these gas bubbles reduce electrical conductivity in the interelectrode gap. When the ECM process is hybridized with a laser, additional synergistic effects are observed. The laser induced temperature elevation takes place in the machining gap depending on the electrolyte flow-field. It has been reported that for the tool-based laser-ECM configuration, the simulated temperature (maximum) in the ECM process (10 V, t_on_ 10 µs, 50% duty cycle) is observed to be 303 K. For the laser-ECM process, the laser irradiation causes localized heating of the workpiece surface with simulated temperatures (considering fluid dynamics) ranging from 325 to 608 K for the average laser power ranging from 2 to 20 W^21^. These temperatures are not sufficient for direct (laser based) material removal and thus only assist electrochemical micromachining of Inconel IN718. Furthermore, the electrolyte heating in the interelectrode gap occurs by a combination of convective and conductive heat transfer between the workpiece surface and the electrolyte. This local escalation of electrolyte temperature results in increased activity/kinetics of electrochemical reactions and causes a local elevation of current density. This results in faster electrochemical dissolution of the workpiece material. This is the fundamental principle for design of laser-electrochemical micromachining processes.

As the hybridization of the laser with ECM leads to faster electrochemical dissolution of the workpiece, it also speeds up generation of reaction by-products including hydrogen gas bubbles. While machining, tool rotation is employed to create stable machining conditions in the gap by flushing out used electrolyte and machining by-products. Additionally, the tool rotation improves the flow-field in the gap and prevents temperature accumulation which can lead to adverse effects. Further scaling-down of the tool combined with tool-path planning, allows more complicated shapes to be fabricated at micro-scale with this technology.

**Figure 3:**
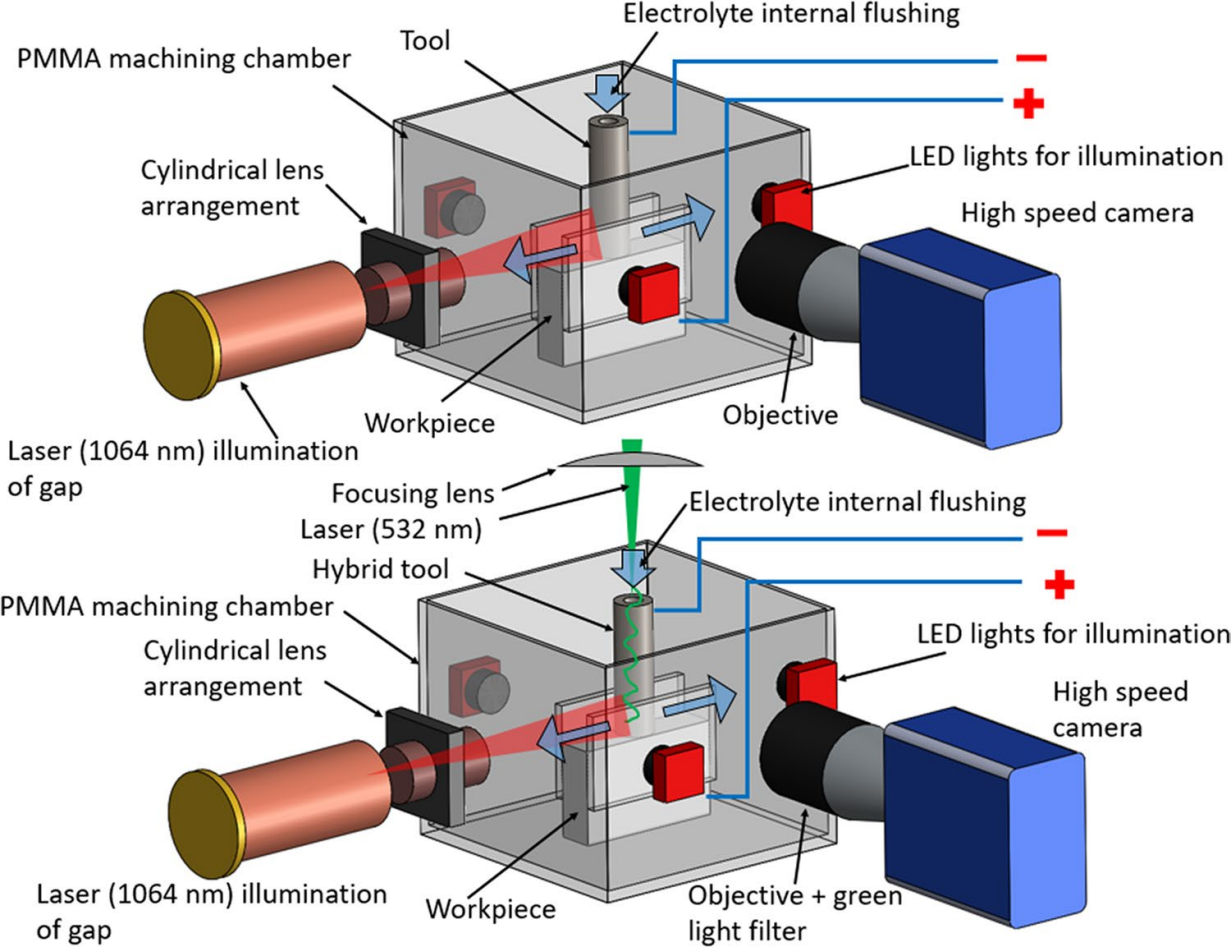
Schematic of the real-time and on-machine high speed imaging experiments on tool-based electrochemical (top) and hybrid laser-electrochemical micromachining process (bottom). An interface was developed in NI LabVIEW software to enable simultaneous start of the process (laser and ECM) as well as the camera recording through an external 5 V TTL trigger. A green-light filter was used in the camera to filter out the green wavelength from the captured images.

**Figure 4:**
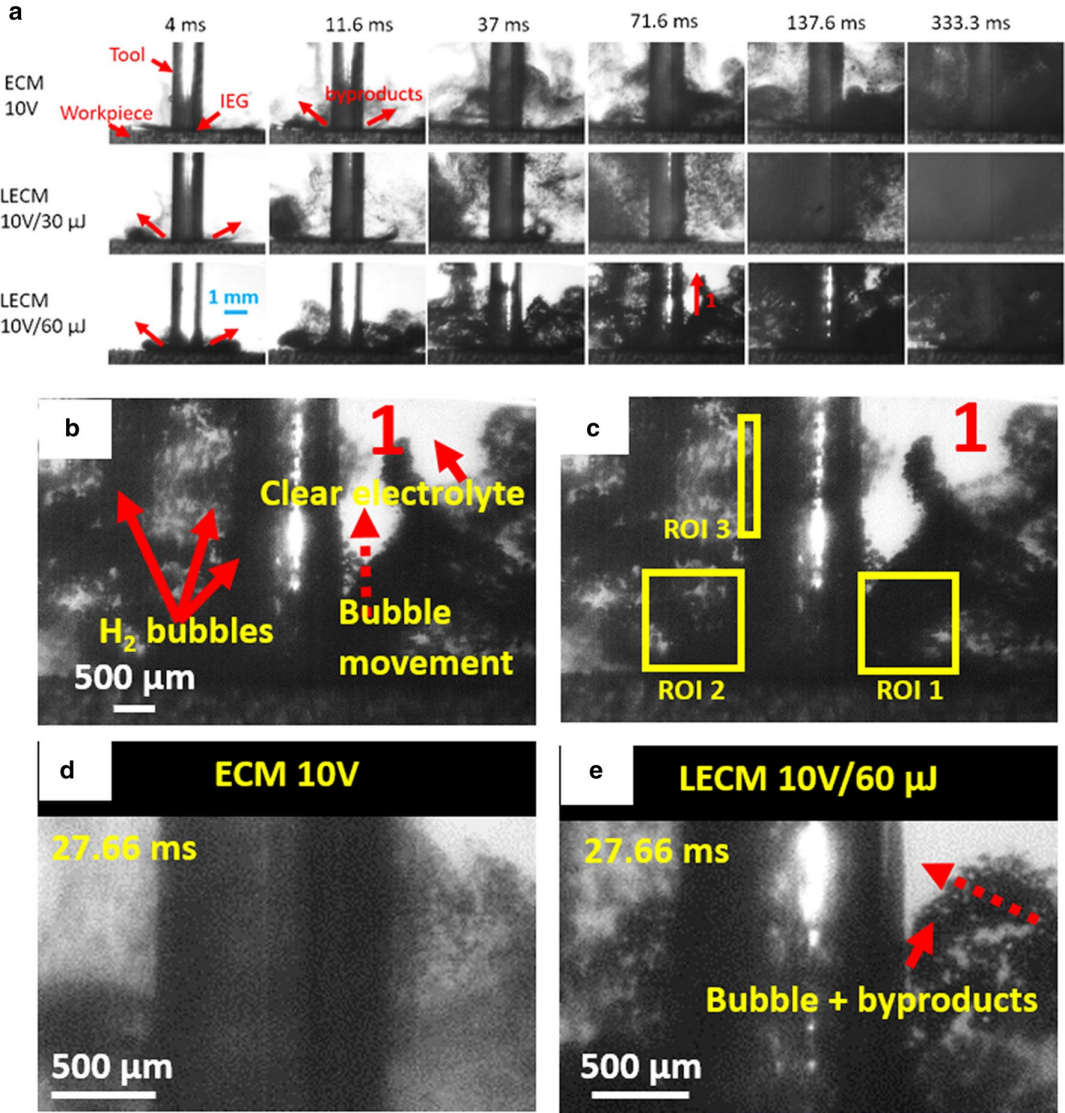
(**a**) Real-time images of by-product and gas-bubble evolution at different frames in ECM and hybrid laser-ECM process at applied voltage of 10 V (**b**) A zoom-in view of the image 1 in (**a**) showing H_2_ bubble movement up along the cathode. (**c**) Selection of regions of interest (ROI) in Matlab interface for monitoring the intensity change with time for quantification of rate of generation of by-products and gas bubbles (**d**, **e**) Comparison of images at the same time-frame for ECM and hybrid laser-ECM process. It is clear that amount of bubble and by-product generation is more in hybrid laser-ECM as compared to ECM at same voltage. All frames shown above are real images subjected to brightness and contrast adjustments to be able to distinguish between by-products and bubbles.

**Figure 5:**
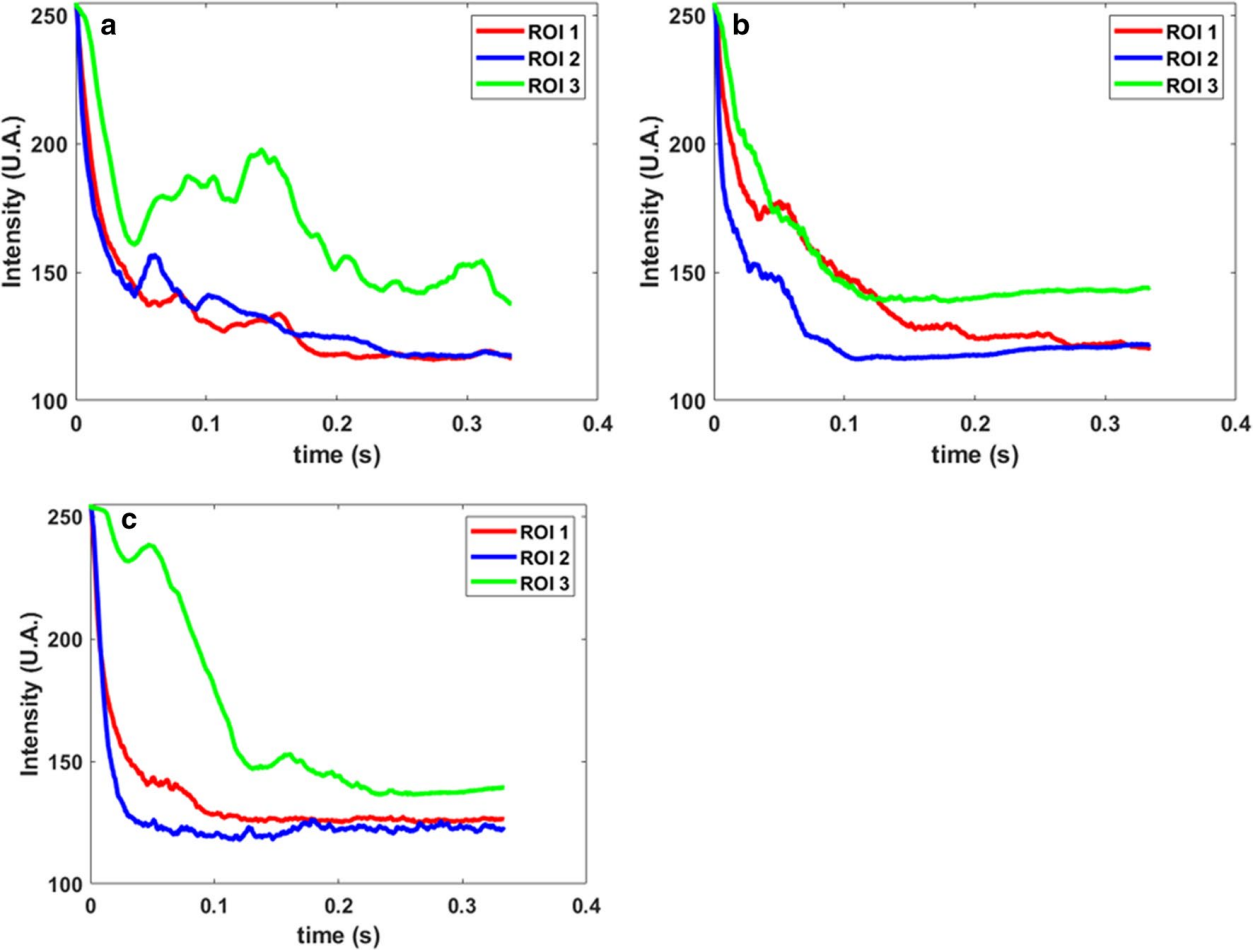
Variation of intensity (in arbitrary unit) in the regions of interest (ROI) (refer Fig. 4c). ROI 1 and 2 are located beside the interelectrode gap on both sides of the tool and indicate the combined rate of generation of reaction byproducts and bubbles. ROI 3 is located as in Fig. 4c and indicates the rate of bubble generation as H_2_ bubbles migrate upwards along the tool (cathode). Intensity variations in (**a**) Tool-based ECM process at 10 V (**b**) Tool-based hybrid laser-ECM process at parameter combination 10 V/30 µJ and (**c**) 10 V/60 µJ.

Figure 3 shows the experimental setup used for real time on-machine observations. The details of the setup are discussed in the methods section. The video observations were conducted using the same tool for all the experiments, so the roughness was constant in all the experiments: *Sa* 0.1816 ± 0.00181 µm, *Sq* 0.2558 ± 0.00717 µm (ISO 16,610–61, L filter 25 µm, S filter 2.5 µm, 20 × objective). Figure 4a shows image sequence close to the interelectrode gap at different times for electrochemical micromachining at 10 V with and without laser. A green light filter is used and hence illumination due to the green laser is not observed. At the beginning (4 ms), the electrolyte is clear for pure ECM as well as for laser-ECM. At 11.6 ms, a strong initiation of electrochemical reaction is observed in case of laser-ECM for both pulse energies in comparison to the pure ECM process. After 71.6 ms, the region in and around the interelectrode gap is filled with by-products including hydrogen gas bubbles for ECM as well as for laser-ECM. In case of laser-ECM, the interelectrode gap is filled quicker and more densely with by-products and bubbles. These results demonstrate that laser-ECM leads to accelerated generation of by-products and bubbles which is an indication of accelerated electrochemical reaction. Figure 4b zooms in on 1 in Fig. 4a, showing gas bubbles around the cathodic tool and also the migration of bubbles up above the cathode thereby supplementing the investigations of Kunieda et al.^17^. For a comparative analysis between ECM and laser-ECM, the change in image intensity around the IEG was evaluated. For this purpose, three Regions of Interest (RoI) were selected in the image as shown in Fig. 4c. RoI 1 and RoI 2 are located on the opposite sides of the tool close to the IEG. These regions of interest indicate a change in intensity in these regions due to all the byproducts (bubbles, reaction by-products, sludge). Thus, all the byproducts pass through RoI 1 and 2 before mixing in the bulk flow. RoI 3 is located close to cathode (tool) and also captures the byproducts passing through this region which consists mostly of hydrogen bubbles in this case. The subsequent discussions will also refer to these RoIs. Figure 4d,e compares images during ECM and laser-ECM at 10 V at a common time frame (27.66 ms). It is evident that more bubbles are observed close to the tool electrode during laser-ECM as compared to ECM alone. The change of intensity over time of different regions of interest (as illustrated in Fig. 4c) is evaluated in Fig. 5. Although, the selection of ROIs is symmetric on both sides of the tool, the observed behavior is not always symmetric. This behavior is due to the fact that at smaller scales it is difficult to realize a high degree of perpendicularity of the tool with respect to the workpiece surface. There is always a slight tilt which causes preferential evacuation of by-products from one of the ROI in some cases. At the start of the experiments, the perpendicularity is set correctly but during repetitive IEG detection by electrical touch on the workpiece surface, a slight tool tilt always comes into picture which results in preferential evacuation of by-products in one of the ROIs. The intensity in RoI 2 drops faster for laser-ECM (Fig. 5b,c) as compared to ECM process (Fig. 5a) which is due to faster generation of by-products and is again an indication of accelerated electrochemical reactions due to laser assistance. The intensity drop in RoI 1 is slightly faster for laser-ECM as compared to ECM process and the observed effects are not as pronounced as seen for RoI 2. This can be due to the fact that, despite maintaining a constant interelectrode gap, the surface roughness of the tool cross-section and the workpiece beneath as well as a slight tilt of the tool may lead to preferential by-product evacuation on the side of tool where RoI 2 is located. Hence, RoI 2 captures the effects better than RoI 1. In case of RoI 3, the intensity fluctuates rather than showing a defined pattern. For the pure ECM process, the intensity drops initially and thereafter exhibits a fluctuating trend. This can be due to the circulation of other by-products close to the tool. For laser-ECM at 30 µJ, the intensity drops initially and then stabilizes. However for laser-ECM at 60 µJ, the intensity change in RoI 3 is slower. This can be explained by the fact that at higher laser pulse energies, the bubbles are rapidly generated and they push each other. Therefore these bubbles are mixed in the bulk flow along with other machining by-products, which does not always allow them to climb along the cathode tool. Hence the intensity drop in RoI 3 is slower for laser-ECM at 60 µJ laser pulse energy. In the later stages of machining, the area of acquisition is completely dark and filled with bubbles and byproducts, therefore the bubbles can rise higher into RoI 3 and hence the intensity stabilizes after dropping.

**Figure 6:**
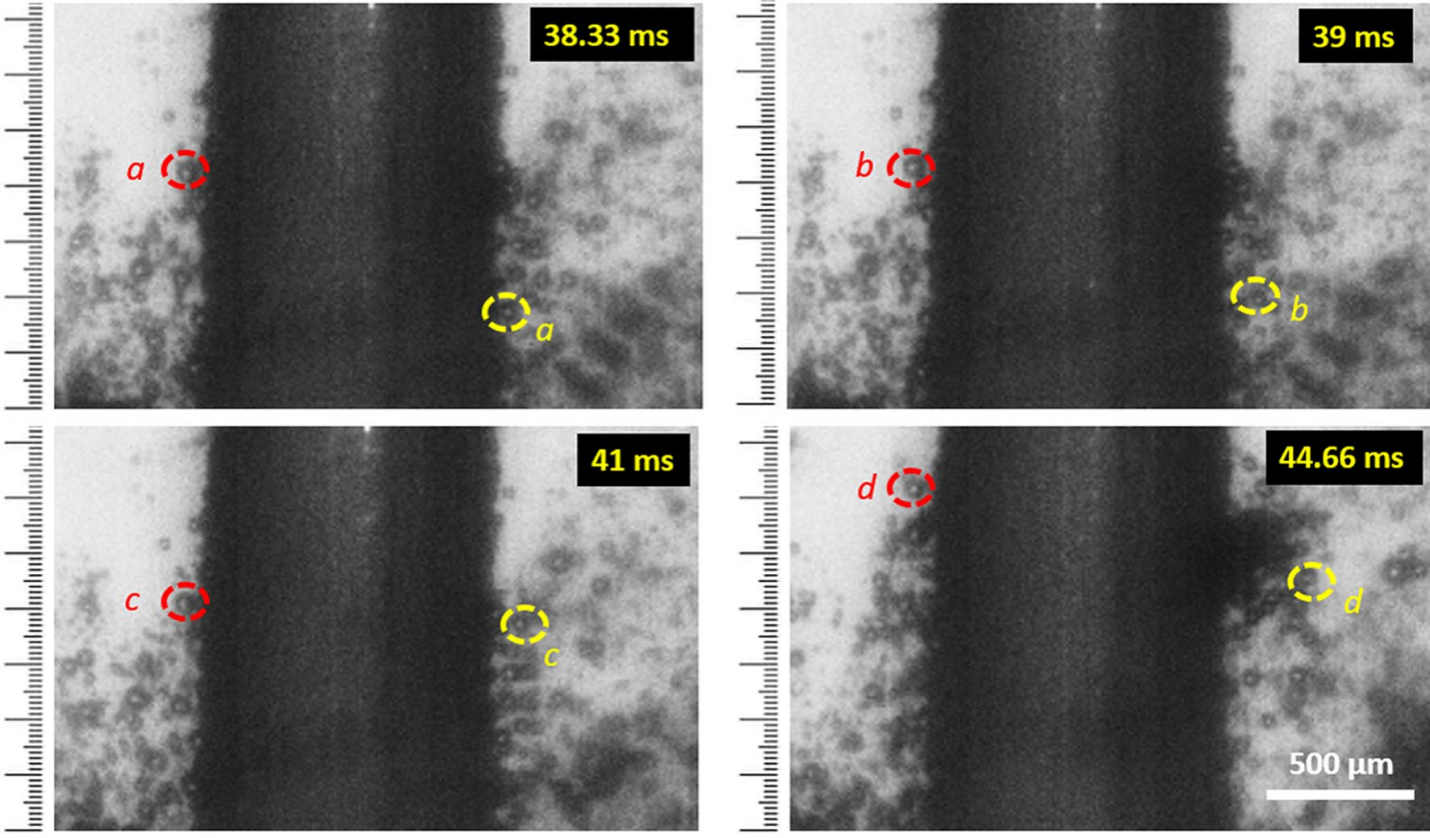
H_2_ bubble motion tracking along the length of tool (cathode) during hybrid laser-ECM process 10 V/30 µJ. The positions of two bubbles were tracked on both sides of the tool at different time-frames and are shown in red and yellow color circles. It is evident that the bubble moves upwards along the length of the tool (cathode) with time.

**Figure 7:**
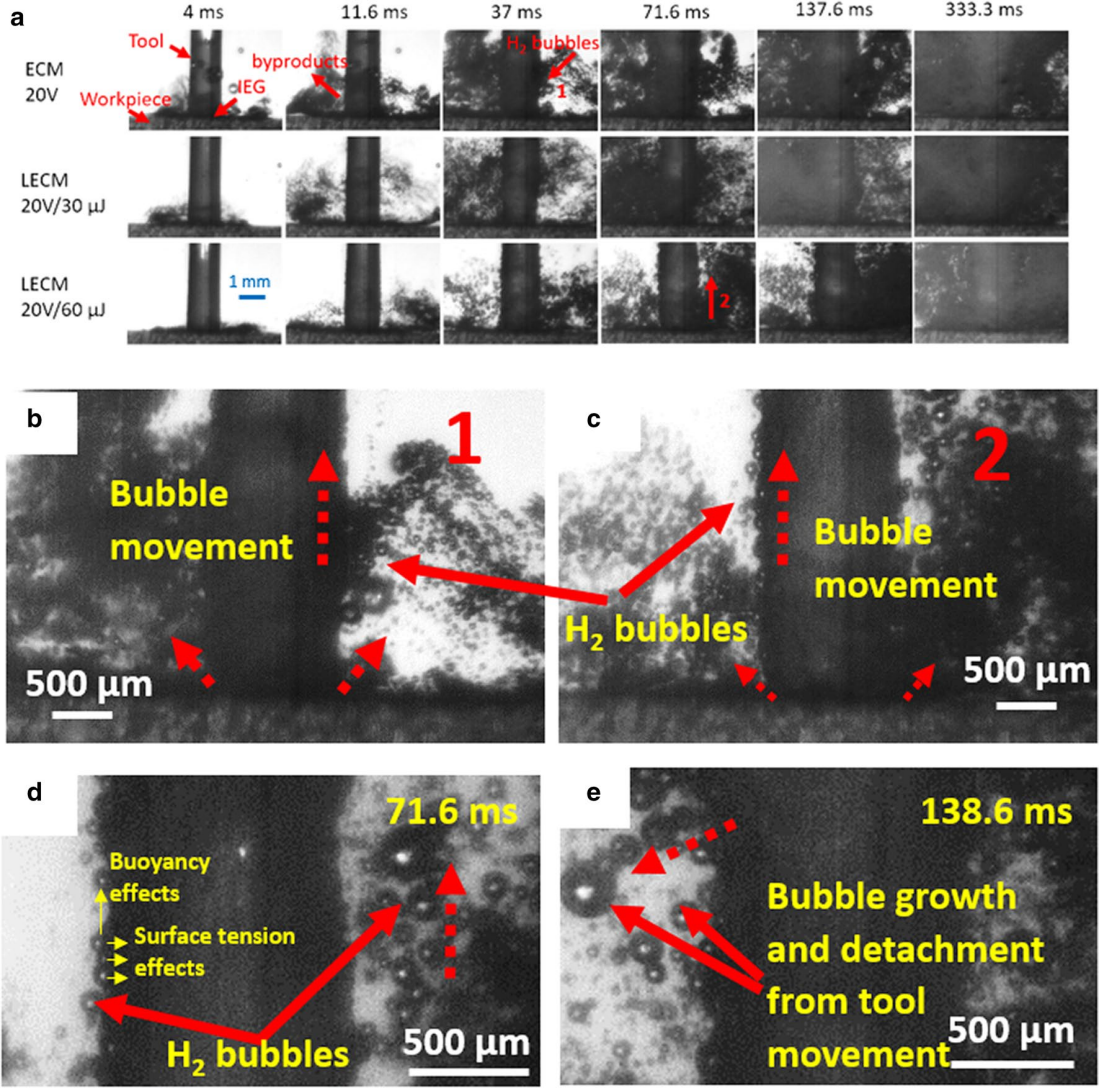
(**a**) Real-time images of by-product and gas-bubble evolution at different frames in ECM and hybrid laser-ECM process at applied voltage of 20 V (**b**) A zoom-in view of the image 1 in (**a**) showing H_2_ bubble movement up along the cathode. (**c**) A zoom-in view of the image 2 in (**a**) showing generation of larger amount of H_2_ bubbles and their movement up along the cathode. (**d**, **e**) Regions of interest in (**c**) are zoomed at two time-frames. Image in (**d**) gives a more clear picture of H_2_ bubble movement up along the cathodic tool. Image in (**d**) shows bubble growth and detachment from the tool. It is clear that amount of bubble and by-product generation is more at 20 V as compared to 10 V and higher in hybrid laser-ECM as compared to ECM process at same voltage. All frames shown above are real images subjected to brightness and contrast adjustments to be able to distinguish between by-products and bubbles. The dashed red arrows indicate the direction of H_2_ bubble movement.

Figure 6 shows images where the motion of two bubbles has been tracked on both sides of the tool as indicated in red and yellow circles during laser-ECM process (10 V/30 µJ). The hydrogen bubbles move vertically upwards along the cathode similar to electrochemical machining. Thermal convection effects (due to laser-ECM) are not pronounced on both sides of the tool in this tool-based laser-ECM configuration. However, thermal convection affects have been observed in beam based laser-ECM methods where stationary or very low electrolyte flow rates are involved and laser beams are focused on the workpiece^11^. As observed in Fig. 6, in the initial frames the hydrogen bubbles are very close (almost attached) to the cathode (tool) and they start detaching as they move upward and tend to mix into the bulk flow. Numerous hydrogen bubbles are observed attached to and around the cathode tool in both Cassie and Wenzel states^23^ depending upon the circumferential surface roughness variation of the tool along its length. Figure 7a depicts an image sequence for ECM and laser-ECM process at a voltage of 20 V. At the initial stage of the process (4 ms), the electrolyte in the acquisition area around the tool is clear and transparent and high illumination can be observed. In the initial frames, no significant difference between process behavior of ECM or laser-ECM is observed. At 11.6 ms, it can be observed that hydrogen bubbles start to appear in ECM process at 20 V whereas in laser-ECM the bubbles are observed in later frames i.e. at 37 ms. Further zooming in on Regions of Interest from Fig. 7a reveals the following.

- Figure 7b depicts zoomed image of a frame (37 ms) during an ECM process at 20 V (marked as 1). The behavior of hydrogen bubbles during ECM is clear in this picture. The hydrogen bubbles are seen to adhere to the cathode and migrate upwards and thereafter start detaching and mix in the bulk flow.
- Figure 7c shows a further zoom in of a frame (71.6 ms) during a laser-ECM process 20 V/60 µJ (marked as 2). The hydrogen bubbles exhibit complicated behavior: some remain attached to cathode due to the surface tension, some rise along the cathode due to buoyancy, some grow in size and some are flushed away in the bulk flow. The region around the tool and IEG is densely occupied with hydrogen gas bubbles which are lined up on the cathode and move upwards. On the opposite side of tool, the bubbles start detaching from tool and mix in the bulk electrolyte flow.
- A further zoom in on Regions of Interest in Fig. 7c is shown in Fig. 7d,e at a time-frame of 71.6 and 138.6 ms, respectively. The hydrogen bubble behavior is more clear in Fig. 7d and is the same as in Fig. 7c. The adherence of bubbles to the cathode tool is observed where the bubbles are in both Cassie and Wenzel states depending on the tool surface roughness. When comparing Fig. 7d,e, it can be observed that the bubbles have grown in size by itself. Due to bubble coalescence they also start detaching from the tool. It is also evident that majority of the gas bubbles grow only close to the cathode and do not seem to grow or coalesce in other regions.

It follows that the behavior of bubbles is not significantly different in tool-based laser-ECM as compared to ECM process. This is because the thermal convection effects are not pronounced outside the interelectrode gap. However, the quantity of hydrogen bubbles and by-product generation was higher than that observed in ECM process.

**Figure 8:**
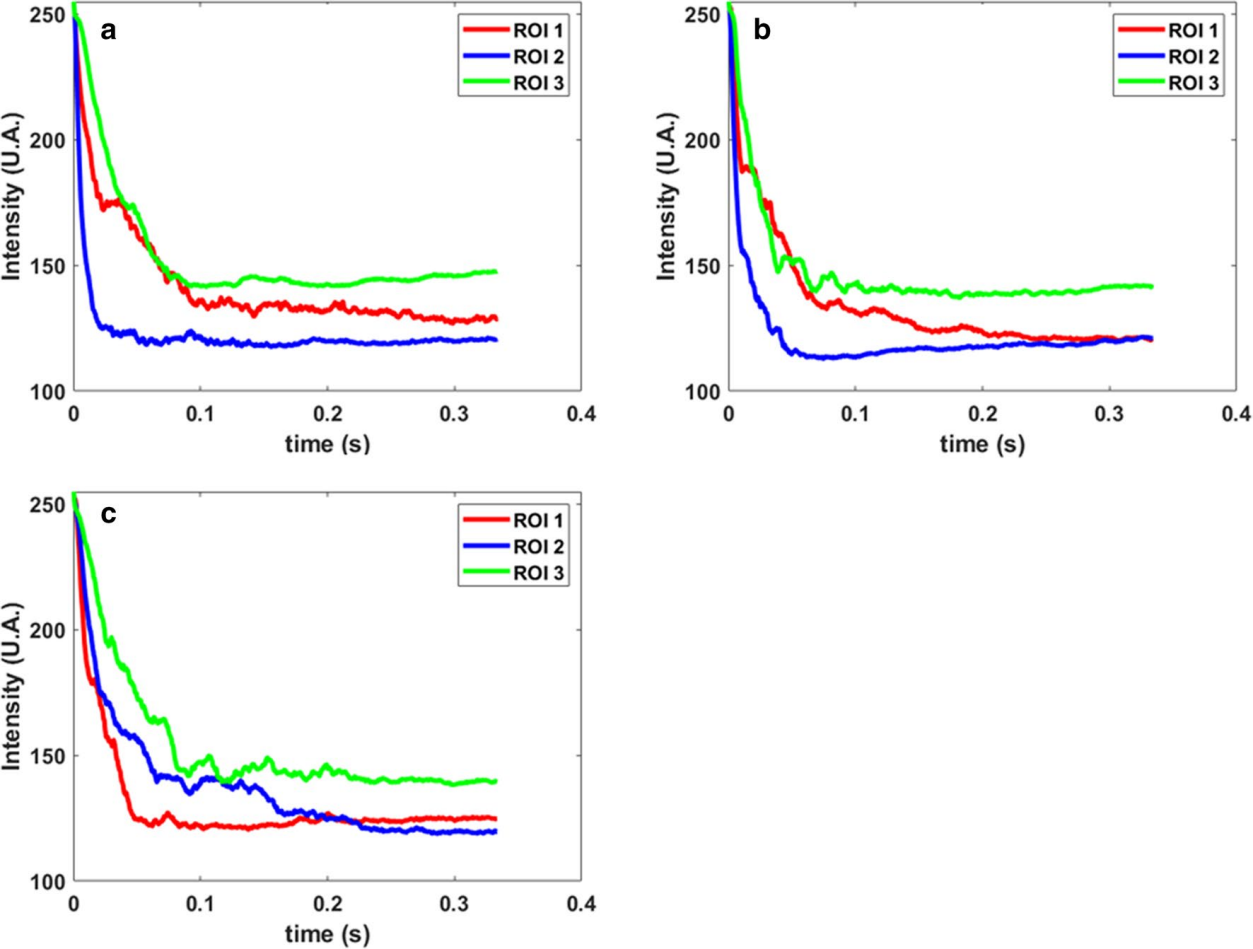
Variation of intensity change (UA: arbitrary units) for ECM and hybrid laser in the regions of interest (ROI) (refer Fig. 4c for locations of regions of interest). ROI 1 and 2 are located beside the interelectrode gap on both sides of the tool and indicate the combined rate of generation of reaction by-products and bubbles. ROI 3 indicates the rate of bubble generation as H_2_ bubbles migrate upwards along the tool (cathode). Intensity variations in (**a**) Tool-based ECM process at 20 V (**b**) Tool-based hybrid laser-ECM process at parameter combination 20 V/30 µJ and (**c**) 20 V/60 µJ.

**Figure 9:**
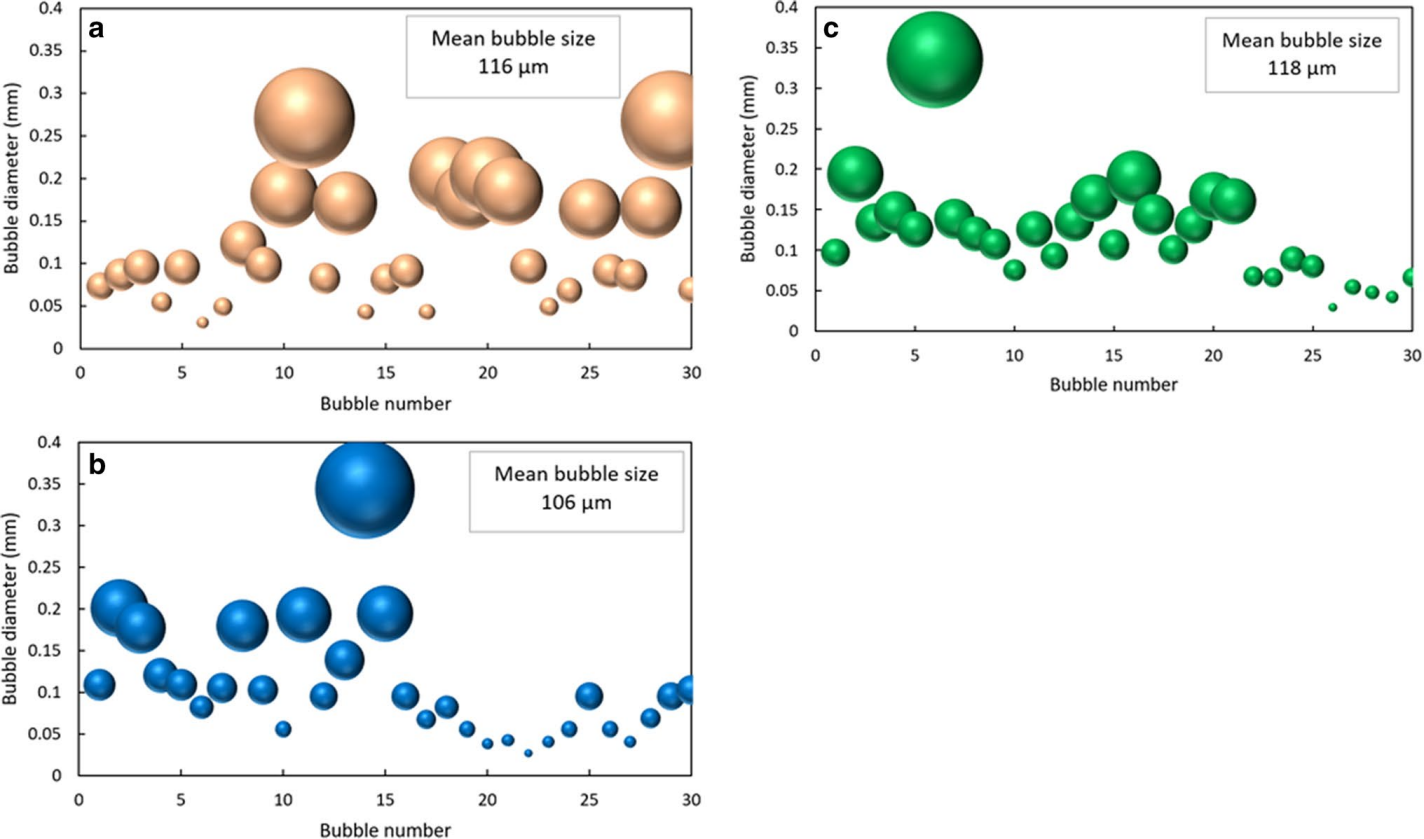
Estimation of diameter of a population of 30 H_2_ bubbles around the cathodic tool at a common time-frame of 71.67 ms for (**a**) tool-based ECM process at 20 V (**b**) tool-based hybrid laser-ECM process at parameter set 20 V/30 µJ (**c**) and at parameter set 20 V/60 µJ. In the bubble plot shown above, the centre of the spheres (bubbles) represent the estimated diameter and the diameter of spheres is proportional to the actual diameter of the bubble. The purpose of spherical representation is to enable a qualitative interpretation of bubble size. The diameters represented above are estimated in PFV ver. 3691 by pixel to distance calibration of image. Only the bubbles around the tool were measured and the smaller bubbles in the bulk flow were not included as they are already being flushed out. Bubbles smaller than 25 µm were difficult to estimate and hence were not included in the measurements.

The plot of Intensity versus time (Fig. 8) indicates that the combined intensity drop of RoI 1 and RoI 2 is faster in laser-ECM process (Fig. 8b,c) as compared to the pure ECM process (Fig. 8a). This also indicates that generation and release of total byproducts in laser-ECM process is faster than that in ECM process alone and is an indication of accelerated electrochemical reaction kinetics by hybridization of ECM with laser. As RoI 3 is taken as an indication of hydrogen bubble generation, the intensity of RoI 3 in laser-ECM (Fig. 8b) drops faster than that in ECM process (Fig. 8a). This indicates a faster rate of hydrogen bubbles crossing through this region of interest. In Fig. 8c also, the intensity of RoI 3 starts dropping in the beginning but later starts fluctuating. This is possibly due to interference of other by-products in RoI 3 which are already circulating in the bulk flow. Measurements of the size of 30 hydrogen bubbles around the cathode tool are shown in Fig. 9 at a common time frame of 71.67 ms. The bubble sizes are measured using a pixel to distance calibration of the image (using PFV v3691 software). With this method, it is difficult to measure bubbles smaller than 25 µm and they were excluded. A significant proportion of bubbles in ECM (Fig. 9a) is bigger in size than those observed in the laser-ECM process (Fig. 9b). It can be clearly observed from Fig. 9 that the number of bubbles with a diameter greater than 0.2 mm is higher in the ECM process as compared to the laser-ECM process. It is also evident that the number of bubbles with 0.2 mm diameter is higher in case of a laser-ECM process with laser pulse energy of 30 µJ as compared to that at 60 µJ. The higher mean bubble diameter value at 60 µJ is due to the larger diameter of one bubble observed in the RoI. This can be explained by the fact that in the pure ECM process, the bubbles get more time to stay at the cathode surface and thereby have time to grow in size and coalesce. On the other hand, in the laser-ECM process, the bubbles are continuously pushed further by the fresh bubbles which are rapidly generated and ejected out from the interelectrode gap and hence these bubbles do not get time to grow in the ROIs. Some bubbles migrate up along the cathodic tool and are pushed by new bubbles and thereafter mix in bulk flow. Some bubbles directly go into flow and are unable to climb up the cathode. These bubbles are unable to grow in size and move out of the machining zone together with the electrolyte flow. Furthermore, it is also observed that the experiments of ECM and laser-ECM at 20 V exhibited more bubbles in comparison to those at 10 V. This is because a higher voltage leads to a higher current density and generation of hydrogen gas depends also on current density. The rate of generation is further amplified by assistance of laser which acts as a heat source.

**Figure 10:**
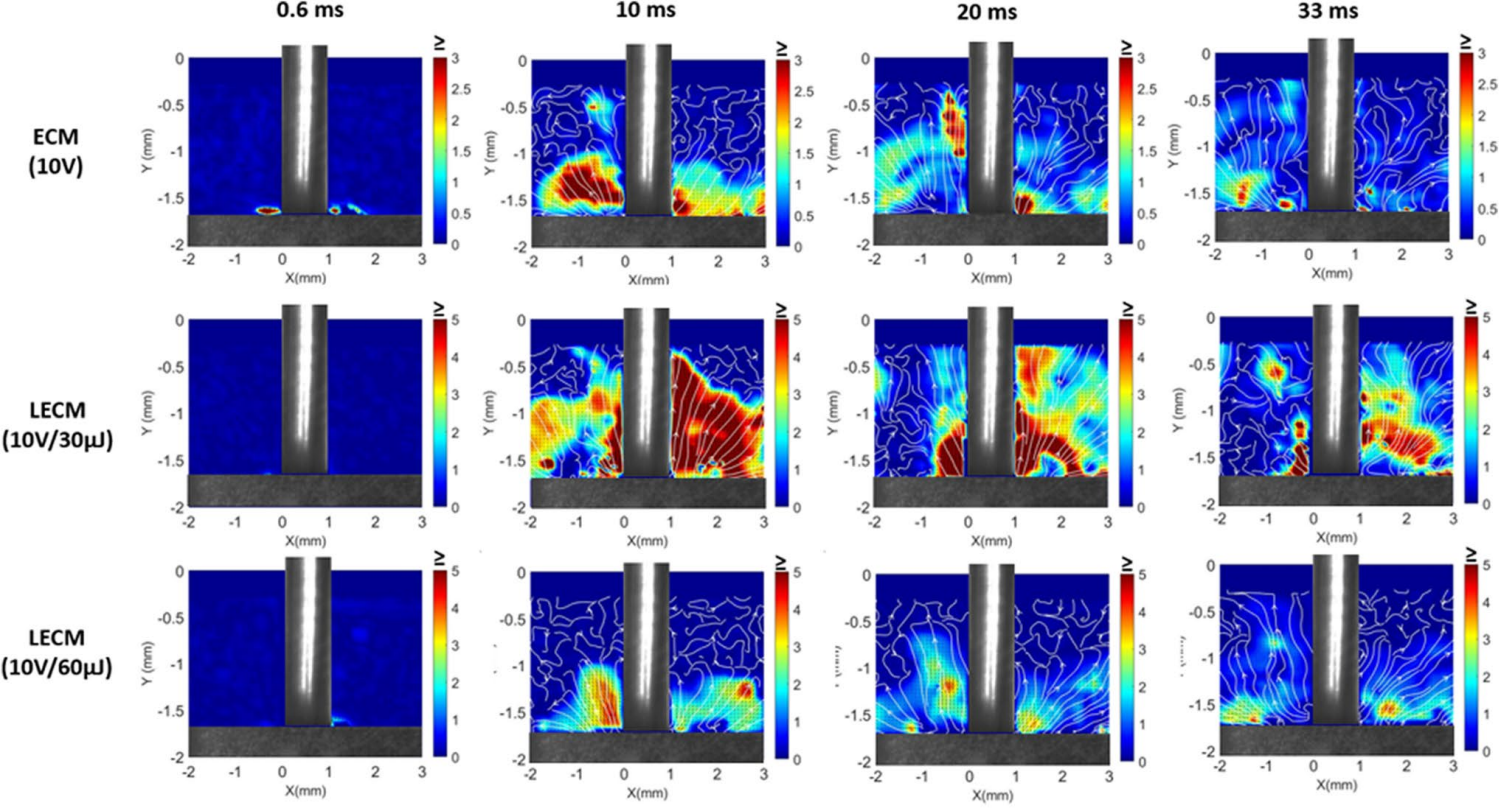
2D LSPIV measurements showing averaged velocity fields during ECM and laser-ECM process at machining voltage of 10 V. The colorbar indicates velocity magnitude in m/s.

Figure 10 shows velocity fields obtained from a 2D LSPIV (large scale particle image velocimetry) analysis for ECM and laser-ECM processes at 10 V. The flow pattern of by-products as well as the velocity magnitude can be observed. In the beginning (0.6 ms), the electrolyte is clear and the initiation of electrochemical reactions can be observed. When comparing the velocity fields of both processes at the same instance, the electrolytic by-products eject out of the interelectrode gap at higher velocity during laser-ECM (at 10 V/30 µJ). This is an indication of the higher reaction rates in the laser-ECM process. A strong initiation of the electrochemical reaction can be observed. It can also be observed from Fig. 10 that for ECM and laser-ECM at 10 V/30 µJ, streamlines are well defined in the starting frames (10, 20 ms) and become less defined in the latter frames (33 ms). This is due to the start of strong electrochemical reaction in the beginning leading to continuous ejection of reaction by-products from the interelectrode gap which is further promoted by the laser pulse energy (30 µJ). Thereafter the reaction declines gradually due to an increase in interelectrode gap. As the reaction declines, new by-product ejection is suppressed and the already existing by-products keep circulating in the bulk flow leading to less-defined streamlines. In case of laser-ECM at 10 V/60 µJ, it can be observed that initially the effect of the laser is not so pronounced. This is evidenced by the limited ejection of electrolytic by-products from the interelectrode gap. Additionally; the streamlines are less defined in the initial frame and become well defined in the latter frames. These observations for laser-ECM at 10 V/60 µJ are possibly due to two reasons.

- In the tool-based configuration of laser-ECM, the laser is focused inside the spindle close to the entrance of the tool electrode and thereafter it propagates further as the tool serves the dual function of ECM electrode and multimode waveguide for the laser. So at the point of focus inside the spindle, plasma formation occurs at higher pulse energies. This initially limits the laser power reaching the workpiece surface and the effects of laser assistance to ECM are thus not so pronounced. This also leads to delayed start of laser-electrochemical reaction. However, as the electrolyte is continuously being replenished, the effects of plasma are short lived and the laser energy reaches the workpiece surface intermittently leading to pronounced synergistic effects in latter frames which are difficult to analyze with PIV as the acquisition area is completely dark due to excessive generation of by-products and particle movement cannot be traced.
- Adverse conditions in the interelectrode gap (local heat accumulation, localized electrolyte boiling/evaporation) arising due to higher laser pulse energy. In the later frames, these conditions improve due to continuous electrolyte flushing as well as pulsing of process energies.

**Figure 11:**
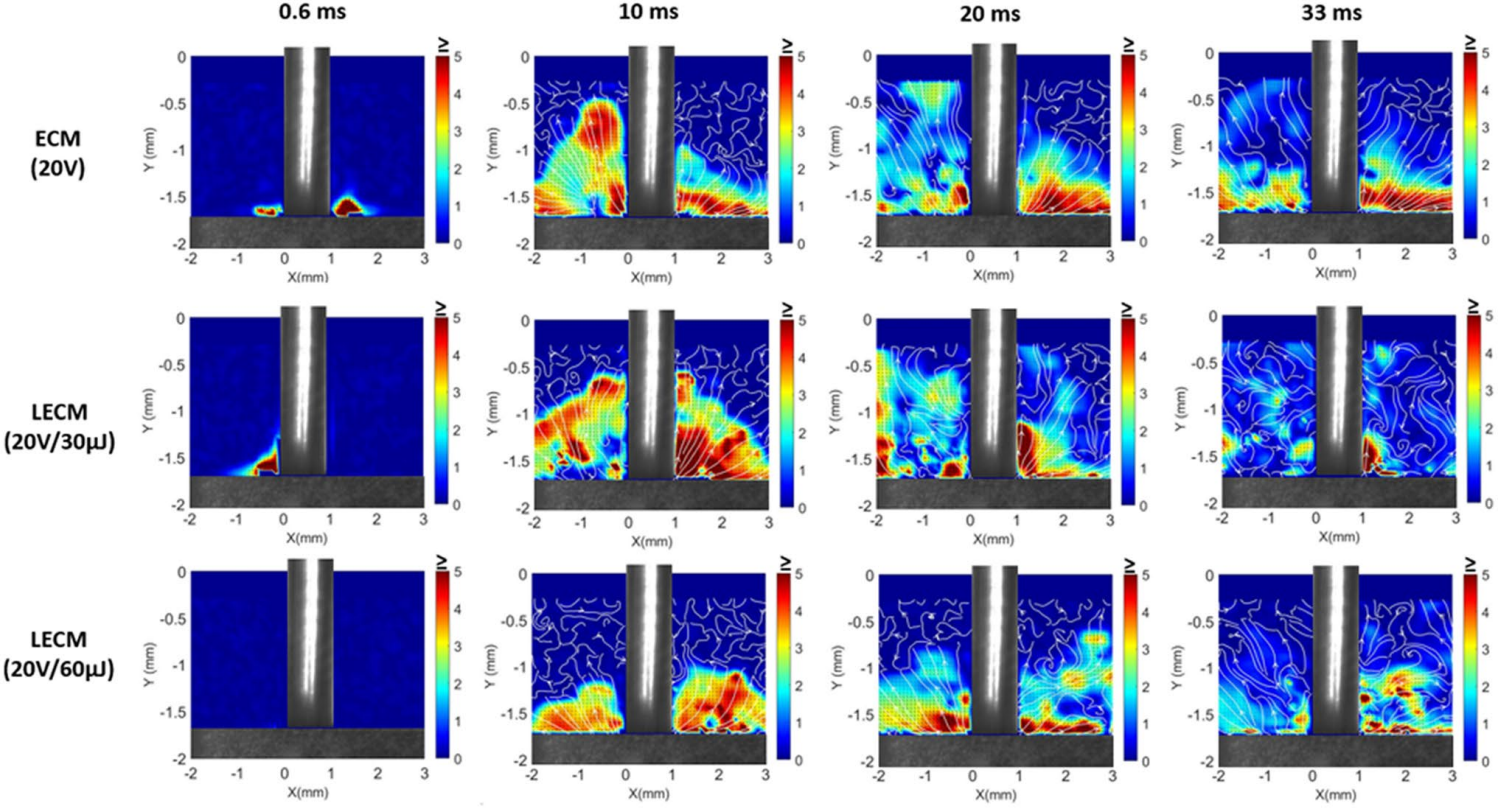
2D LSPIV measurements showing averaged velocity fields during ECM and laser-ECM process at machining voltage of 20 V. The colorbar indicates velocity magnitude in m/s.

For ECM and laser-ECM processes at 20 V, the velocity fields obtained from 2D PIV analysis are shown in Fig. 11. It is clearly seen that the higher machining voltage leads to an increased generation of byproducts and with laser assistance this rate is even further enhanced. The effect of voltage manifests itself in the form of increased current density in the interelectrode gap. Therefore, a strong start of reaction is observed at higher velocities for both ECM and laser-ECM. The streamlines are well defined for ECM process at 20 V due to continuous ejection of by-products from the interelectrode gap and this is an indication that the reaction (material dissolution) continues longer due to higher current density at higher voltage. With laser-ECM at 20 V/30 µJ, there is sudden and accelerated ejection of by-products as an indication of strong start of the reaction due to laser-ECM synergistic effects. For laser-ECM at 20 V/30 µJ the streamlines are well defined in the initial frames (10, 20 ms) and become less defined in latter frames (33 ms) as the dissolution is completed faster due to strong synergistic effects. The by-product generation is reduced and the ejected by-products keep circulating in the studied region. This phenomenon is also observed for laser-ECM at 20 V/60 µJ but less pronounced than that at 20 V/30 µJ because initially it is purely due to voltage effect rather than laser effect as at 60 µJ the laser effect is not pronounced initially due to aforementioned reasons. The streamlines continue to stay well defined in all the frames indicating ejection of by-products for longer time and indicate that material dissolution sustains longer initially due to voltage and thereafter due to the synergistic effect of laser and ECM.

## Conclusions and outlook

In general a hybrid laser-ECM process using a tool-based configuration is interesting as it allows a broadening of the material processing window, machining of materials with conductivity variations, application of laser and ECM coaxially and concurrently at higher machining depths, facilitation of electrochemical dissolution by structural weakening of passivation layer, accelerated material removal and improved surface quality in specific processing window. Till date, the mechanisms of process interaction and synergistic effects are not completely understood.

The on-machine high speed imaging and PIV investigations presented here show preliminary but successful insights into some of the phenomena such as by-product and hydrogen gas bubble generation as well as their physical and flow behavior around the interelectrode gap. These observations have also indirectly indicated the enhancement of rate of electrochemical reaction during hybrid laser-ECM process in comparison to the pure ECM process. The accelerated generation of by-products and hydrogen gas bubbles will affect the electrical conductivity of the electrolyte ultimately leading to deterioration of part quality. This also necessitates for continuous internal flushing as well as tool rotation in this hybrid laser-ECM process. The current work also shows the behavior of by-products and gas bubbles around the tool close to the interelectrode gap. The flow behavior and by-product generation is further clarified by means of quantitative results from 2D PIV study.

Different behaviors regarding hydrogen gas bubbles have been identified. (1) The hydrogen gas bubbles in hybrid laser-ECM do not have time to grow in size as they are continuously being pushed by newly generated bubbles. On the other hand, this is not the case in a pure ECM process where the bubbles are relatively bigger in size. (2) The hydrogen gas bubbles migrate upward along the tool length and in this case the behavior is similar for both laser-ECM and ECM processes as there are no thermal convection effects on both sides of the tool. (3) Higher laser pulse energies tend to affect the process adversely. This is possibly due to plasma plume formation (and possibility of electrolyte boiling) at the point of focus inside the machining head leading to reduced laser fluence available at the workpiece side. By pulsing both process energy sources (laser and ECM), the effects of excessive heat accumulation in the interelectrode gap can be minimized but the initiation of a synergistic effect gets delayed. (4) Additionally, at higher voltage the reaction is initiated faster but the synergistic effects are delayed (observed in later frames) which indicates a voltage dependence rather than a laser dependence.

In future works, more sophisticated and improved real-time on-machine observations could facilitate development of mechanism based hybrid laser-ECM process which will lead to production of high quality parts with improved metallurgical or functional properties. These observations will also facilitate multidisciplinary finite element simulations to better predict the processing response and minimizing the product lead-time. For manufacturing industries, this research is a direction towards Industry 4.0 where these high speed cameras can be used in closed loop control to make hybrid processes more deterministic.

## Methods

### Hybrid laser-electrochemical micromachining

For the experiments on hybrid laser-electrochemical micromachining, an in-house developed prototype hybrid machine-tool was used. Figure 1 shows the developed hybrid micromachining setup with major peripherals^13,14^. The hardware consists of a granite gantry-type frame, a green laser module alongwith dedicated optics, motion stages, a hybrid machining head, an electrolyte supply and handling system, a pulsed voltage source, a machining cell and a NI LabVIEW based control unit. The high frequency voltage pulses for ECM process are derived by switching a DC voltage source using an NMOS switch. The inter-electrode gap is set manually using an electric-touch procedure. A pulsed laser source from Spectraphysics-Newport VGEN-G-HE-30 (avg. power 30 W, ns pulsed, max. pulse repetition rate 1500 kHz, max. pulse energy 180 µJ, wavelength 532 nm) is used as a second energy source. The control and data acquisition is realized with a NI CompactRIO system. A 200 g/l* aq.* NaNO_3_ solution was used for all the experiments. For the on-machine observations, the voltage pulse parameters were maintained constant (pulse on time 10 µs and duty cycle 50%) in all the experiments.

### Workpiece sample preparation

Inconel IN718 alloy (composition: 55Ni–21Cr–5Nb–3Mo wt%) samples of 20 × 20 × 3.17 mm were used for experiments. The samples were polished on three sides with polishing papers of P500 and P800 grits. After polishing, the samples were subjected to ultrasonic cleaning in de-ionized water (8 MΩ cm). Since, IN718 is not optically transparent, a sandwich workpiece was designed for real time observations during machining as highlighted in the work of Natsu et al.^24^. The IN718 workpiece was sandwiched between two glass plates of 20 × 20 × 3 mm. This enabled flow confinement to lateral direction and the camera could clearly observe the phenomenon close to the inter-electrode gap.

### High speed imaging

In order to perform real time observation of hybrid laser-ECM process, a dedicated setup was developed as shown in Fig. 3. A Photron SA3 high-speed camera was used in combination with Tamron N-AFD 2 × teleconverters and 3 extension tubes and a Nikon NIKKOR 105 mm macro lens. A high pass interferometric filter was additionally placed in front of the camera objective for the experiments performed with laser-ECM to eliminate the laser contribution from the images. The camera unit was mounted on a vibration free flat platform from Thorlabs for specific studies performed in this work. The recordings were carried out at a framerate of 3000 fps and a shutter time of 0.33 ms. An image processing code has been written in Matlab to analyze the intensity variations (as an indicator of by-product/bubble generation) as a function of time in defined Regions of Interest (RoI). To obtain intensities in RoIs (Figs. 5 and 8), an image of the RoI without bubbles/by-products is subtracted from each image (background removal), then the intensity levels in the RoIs are averaged. Removing the background allows to have a better contrast in the images and to increase the signal to noise (S/N) ratio. No further post-processing is conducted on the images. An example is presented here in Figure S1 (refer supplementary material) via the histogram of the grayscale intensities of an image, for which the contrast, defined as the difference between the maximum and the minimum grayscale level is equal to 163. The tool rotation was not employed in order to prevent wiping out of by-products from the camera Field of View (FoV). Experiments were conducted at very low flow rates so as to be able to visualize the by-product and bubble generation. The visualization was performed close to the interelectrode gap. Three LED lights and a red pilot laser were employed to reach sufficient illumination and to avoid image flickering during recordings. An interface was developed in NI LabVIEW software to enable simultaneous start of the process (laser and ECM) as well as the camera recording via an external 5 V TTL trigger.

### Large scale particle image velocimetry (PIV)

Large scale particle image velocimetry (LSPIV) has been conducted in order to measure the velocity of by-product while travelling into the flow after production. Unlike Particle Image Velocimetry, the LSPIV measures the velocity of dispersion structures and not of individual particles. However, the two methods are based on the same principle of image cross-correlations. In particular, to obtain the velocity of the structures formed by the by-products, the images acquired via high speed imaging have been initially filtered, to remove background noise, then a mask is applied to limit the Region of Interest. The software DAVIS 8.4 from LaVision has been used to determine the velocity of the by-product structures. The parameters used for the PIV processing are reported in Table 1.

**Table 1:** Parameters used for the PIV processing.

Parameter	Value
Initial window size	96 × 96 px^2^
Final window size	32 × 32 px^2^
Windows overlap	75%
Minimum cross correlation peak ratio ${Q}_{min}$	1.5

It is important to underline that, due to the complexity of the observed phenomenon, the presence of bubbles in the Field of View, the rapid change of the image contrast and the high concentration of the particles composing the structures, the obtained velocity shows a considerable uncertainty. Nevertheless, they qualitatively faithfully present the kinetics of the clouds generated by the of by-products and can be used to understand the physical mechanisms associated to their production.

## Supplementary information

Supplementary Figure S1.
