# Supplementary material for: Real-time on-machine observations close to interelectrode gap in a tool-based hybrid laser-electrochemical micromachining process
Source: Sci Rep. 2020 Oct 8;10:16797. doi: 10.1038/s41598-020-73821-7 (PMC7544913; doi:10.1038/s41598-020-73821-7)
Supplement: Supplementary file 1 — Supplementary Figure S1. [file 41598_2020_73821_MOESM1_ESM.docx]

**Real-time on-machine observations close to interelectrode gap in a tool-based hybrid laser-electrochemical micromachining process**

Krishna Kumar Saxena^1,3^, Xiaolei Chen^1,4,5^, Maria Rosaria Vetrano^2^, Jun Qian^1,3^, Dominiek Reynaerts^1,3^

^1^Micro-& Precision Engineering Group, Manufacturing Processes and Systems, Department of Mechanical Engineering, KU Leuven, Belgium

^2^Heat and Mass Transfer Group, Division of Applied Mechanics and Energy Conversion, Department of Mechanical Engineering, KU Leuven, Belgium.

^3^Member Flanders Make, Leuven, Belgium.

^4^School of Electromechanical Engineering, Guangdong University of Technology, Guangzhou, China.

^5^Guangzhou Key Laboratory of Nontraditional Machining and Equipment, Guangzhou, China.

Corresponding author: dominiek.reynaerts@kuleuven.be


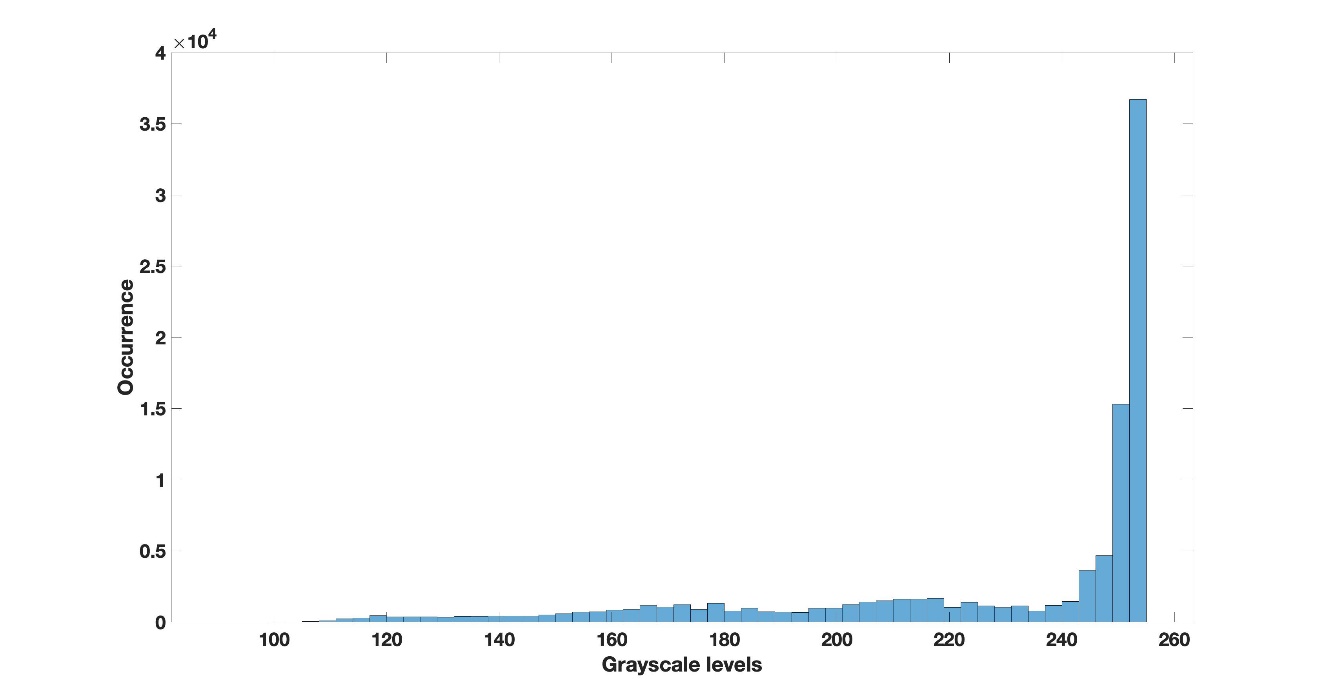


**Figure S1:** Exemplar histogram of the grayscale intensities of an image, for which the contrast, defined as the difference between the maximum and the minimum grayscale level is equal to 163.
